# Swimming behavior indicates stress and adaptations to exercise

**DOI:** 10.3389/fphys.2024.1357120

**Published:** 2024-02-26

**Authors:** Sen Yu, Lantao Liu, Min Li, Siyan He, Yang Hu, Shichao Sun, Yizhen Yan, Fangfang Zhao, Xiangrong Cheng, Jia Li, Feng Gao, Yong Liu, Xing Zhang

**Affiliations:** ^1^ Key Laboratory of Ministry of Education, School of Aerospace Medicine, Fourth Military Medical University, Xi’an, China; ^2^ Department of Medical Electronics, School of Biomedical Engineering, Fourth Military Medical University, Xi’an, China; ^3^ Chengdu Techman Software Co., Ltd., Chengdu, China; ^4^ Department of Rehabilitation, Air Force Medical Center, Beijing, China

**Keywords:** swimming behavior, cardiac adaptation, exercise, trajectory tracking, stress

## Abstract

**Introduction:** Behaviors of swimming rodents are not uniform, exhibiting large variations, which may underlie the individual differences in swimming exercise-induced benefits. The study aimed to monitor individualized swimming behavior and evaluate its biological significance.

**Methods:** A swimming tank which can monitor individualized rodent swimming behavior during exercise was established. A total of 45 mice were subjected to swimming training for 1 month (1 h per day) and the swimming behaviors of each mouse were recorded.

**Results:** The swimming behaviors of mice displayed considerable variations in aspects of distance, velocity, and area preference. For example, nearly one-third of mice preferred to swim in central area and most of the mice exhibited an even area distribution. Long-term exercise training improved cardiac systolic function and decreased blood pressure in mice, but hardly changed swimming behaviors. Analyses of the relationship between swimming behavior and cardiovascular adaptations to exercise training revealed that swimming behavior indicated the biological effects of swimming training. Specifically, mice which preferred swimming at the central zone or were trainable in behavior during 1-month training exhibited better outcomes in cardiac function and blood pressure post long-term exercise. Mechanistically, a centralized swimming behavior indicated a smaller stress during exercise, as evidenced by a milder activation of hypothalamic–pituitary–adrenal axis.

**Discussion:** These results suggest that swimming behavior during training indicates individualized adaptations to long-term exercise, and highlight a biological significance of swimming behavior monitoring in animal studies.

## 1 Introduction

It is well recognized that exercise exerts pleiotropic benefits on overall health, fitness, and quality of life, which behaves like an endogenous “medkit” with unlimited refill within the body (Wu et al., 2021; Zhang and Gao, 2021; Lou et al., 2022; Valenzuela et al., 2023). However, the science base for exercise and health has lagged behind other important issues such as smoking (Kohl et al., 2012). Although deciphering the underlying mechanisms governing exercise-induced benefits has attracted much more attention nowadays, the precise molecular signaling processes leading to structural and functional adaptations to exercise are insufficiently understood. Thus, animal studies exploring the underlying mechanisms of exercise-induced benefits are tremendously helpful for understanding exercise physiology and furthering exercise translation.

Since rodents have the innate ability to swim and swimming requires less expensive and less elaborate equipment allowing a large number of rodents to swim readily and simultaneously, swimming exercise is one of the mostly used models of moderate-intensity aerobic exercise in animal studies (Jones, 2007; Seo et al., 2014; Hou et al., 2019; Poole et al., 2020; Li et al., 2022; Yan et al., 2022). It recruits a large volume of muscle mass and induces extensive cardiovascular adaptations, including physiological cardiac hypertrophy and blood pressure decline (Hou et al., 2019; Zhang and Gao, 2021; Li et al., 2022; Valenzuela et al., 2023). However, there are still several challenges which may lead to misinterpretation of animal experiments. Firstly, swimming duration is usually used as a parameter in quantifying exercise intensity, while the workloads of swimming rodents are not uniform (Gobatto et al., 2001; Guo et al., 2020; Wang et al., 2020; Hastings et al., 2022). There are considerable individual differences in swimming behavior among animals, leading to large variations in exercise intensity (Ryder et al., 1999; Ren et al., 2000; Kirchhof et al., 2003). For instance, some animals do not demonstrate continuous swimming behavior but resort to floating, diving, or bobbing behavior, while other animals are more enjoyable and active (Ryder et al., 1999; Jones, 2007; Poole et al., 2020). Secondly, forced swimming exercise produces stress which varies among rodents, although evidence has shown that the stress is mild and does not induce observable adverse effects when the procedure is well handled (Contarteze et al., 2008; Liu et al., 2018; Song et al., 2022). Finally, it is well established that swimming exercise induces extensive biological adaptations, but how swimming behavior itself adapts to long-term exercise is unknown (Bostrom et al., 2010; Egan and Zierath, 2013; Spaulding and Yan, 2022). Thus, there is an urgent need to establish a method to monitor rodent swimming behavior during exercise for assessment of individualized workload, stress, and performance.

Here, we established a swimming tank which can monitor individualized rodent swimming behavior during exercise using a camera-based trajectory tracking strategy. The distance, velocity, and location of each mouse can be quantified in real-time. A total of 45 mice were subjected to swimming exercise for 1 month. Analyses of the relationship between swimming behavior and cardiovascular adaptations to exercise training revealed that swimming behavior indicates the biological effects of swimming training. These findings highlight a biological significance of swimming behavior monitoring in animal studies.

## 2 Materials and methods

### 2.1 Animals

C57BL/6 mice (8 weeks of age) were obtained from the Experimental Animal Center of Fourth Military Medical University (China). Mice were housed in a temperature-controlled room with a 12 h light/12 h dark cycle and given unrestricted access to water and food.

### 2.2 Exercise protocol and swimming behavior monitoring

The swimming training protocol was modified from a previously published procedure (Liu et al., 2015; Hou et al., 2019). Briefly, C57BL/6 mice swam once daily for 6 days per week for 4 weeks. All biological parameters were detected at 24 h after the last training session. The first day of swim training consisted of two 10-min sessions separated by a minimum of 4 h. Sessions were then progressively increased to 60 min/d over 1 week. A total of 45 mice were subjected to swimming training using a custom-developed swimming tank. The tank was constructed by Chengdu Techman Software Co., Ltd. (Chengdu, China). Mouse swimming was performed in an open area of 80 cm (length) ×50 cm (width). The water depth was 15 cm with a water temperature of 35°C.

**FIGURE 1:**
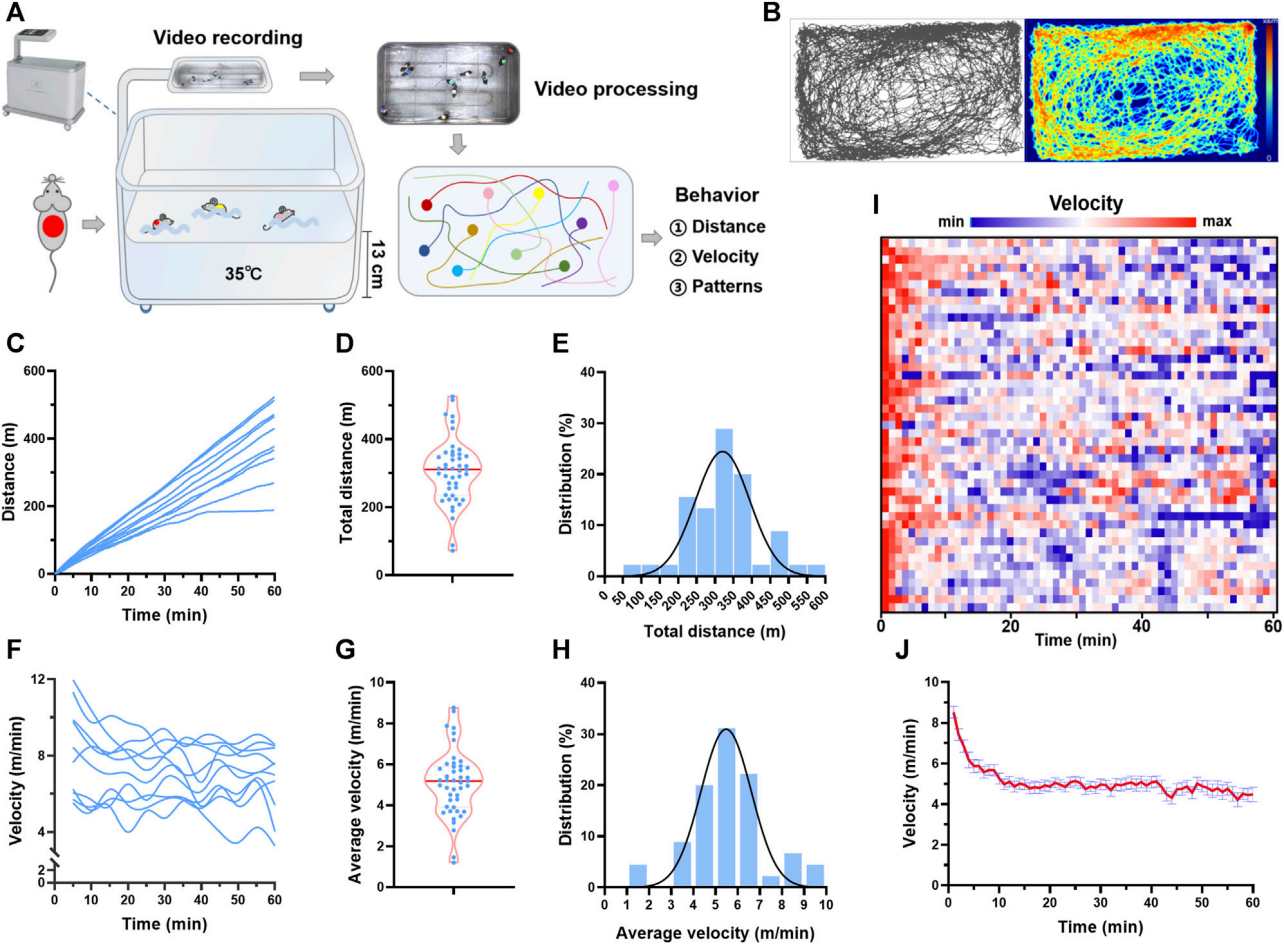
Trajectory tracking revealed individual differences in behavior during swimming. **(A)** Real-time video analytics was used to monitor the locomotor activity of swimming mice using a custom-established rodent swimming tank. **(B)** Typical trajectory plots of one mouse during a session of swimming training. The color coding the time spent in the coordinate. **(C)** Typical distance traces of 10 mice in one session of swimming training. **(D)** Total distance distribution of 45 mice. **(E)** Total distance histogram of 45 mice. **(F)** Typical velocity traces of 10 mice in one session of swimming training. **(G)** Average velocity distribution of 45 mice. **(H)** Average velocity histogram of 45 mice. **(I)** Heatmap showing the velocity along with time of 45 mice during one session of swimming training. **(J)** Quantified velocity of 45 mice during one session of swimming training. n is indicated in each panel.

Mice were randomly divided into groups, with 8–10 mice in a session of swimming exercise, and marked with different colors using stickers (Figure 1A). The camera recorded the behavior of mice simultaneously with a multi-objective color-picking image recognition algorithm. Videos are captured in MP4 format with a resolution of 1280 × 720 pixels and 10 frames per second. The video files are processed frame by frame, and the position of each mouse along with time was recorded. The distance, velocity, area preference, and status of swimming were calculated. The distance within t seconds is calculated by $\sum_{i=1}^{t}\sqrt{{\left(x_{i+1}-x_{i}\right)}^{2}+{\left(y_{i+1}-y_{i}\right)}^{2}}$ ; the instantaneous speed at the time of i second is calculated by $\sqrt{{\left(x_{i+1}-x_{i}\right)}^{2}+{\left(y_{i+1}-y_{i}\right)}^{2}}$ ; the average speed within t seconds is calculated by $\frac{\sum_{i=1}^{t}\sqrt{{\left(x_{i+1}-x_{i}\right)}^{2}+{\left(y_{i+1}-y_{i}\right)}^{2}}}{t}$ ; where x and y indicate the real-time coordinate of each mouse. For area preference, the swimming zone was divided into 16 equal parts, the central 4 areas were defined as central zone, and the rest areas were defined as peripheral zone. The regional preference was calculated based on the coordinates of each mouse. For swimming status, inactive swimming was defined as the instantaneous velocity <1 cm/s, otherwise the mouse is active.

### 2.3 Grip strength

A grip-strength meter (BIO-GS3; Bioseb, Pinellas Park, FL, United States) was used to measure the grip strength (four limbs) of the mice. The mice were put on the grid, and their torsos were kept horizontal. Then the tails of mice were gently pulled back and their torsos were parallel to the grid to eliminate the interference of back strength. The grip strength of each mouse was measured 10 times continuously to calculate the maximum and average values.

### 2.4 Fasting blood glucose

Mice were fasted overnight (12 h) and tail vein blood was used for glucose measurement with an ACCU-CHECK blood glucose meter (Roche Diagnostics, Mannheim, Germany).

### 2.5 Blood pressure

Blood pressure was measured by the tail cuff (BP-98A, Softron). Each mouse was measured 3 times and the average value was calculated and recorded.

### 2.6 Measurement of circulating adrenocorticotropic hormone (ACTH), cortisol, and corticosterone

Animals were sacrificed immediately after 1 h of acute swimming exercise for blood collection. The plasma ACTH, cortisol and corticosterone levels were tested using ELISA kits (Cloud-Clone Corp., Wuhan, China) according to manufacturer’s instructions.

### 2.7 Cardiac function

Mice were anesthetized by 2% isoflurane and ventricular function was determined by an echocardiographic imaging system (Vevo^®^2100, Visual Sonics, Toronto, ON, Canada). Two-dimensional echocardiographic views of the mid-ventricular short axis were collected at the section of the papillary muscle tips below the mitral valve. The left ventricular ejection fraction, fractional shortening rate, cardiac output, and stroke volume were calculated.

### 2.8 Open field test

Each mouse was placed in a 40 × 40 × 40 cm black plastic open field so that it was free to explore for 10 min. The central zone was defined as a central 20 cm × 20 cm square, and the other region was defined as the peripheral zone. Between all test sessions, 75% alcohol was used to remove odors from the open field test apparatus. The animal behavior experiment video analysis system (JLBehv-OFG-1, China) was used to record and analyze the trajectory and related data of each mouse in the open field.

### 2.9 Statistics

Data are expressed as mean ± SEM. GraphPad Prism 8.2.1 software, Excel and OriginPro (v.9.8) were utilized in data presentation. Statistical analyses were conducted using SPSS 26 (IBM, Armonk, NY). Kolmogorov–Smirnov normality test was used to analyze the normal distribution of the data. The frequency distribution histogram of total distance, average velocity and central distance/total distance was fitted using simple Gaussian algorithm. Two-tailed paired t-tests were applied to compare the mean value between pre-training and post-training. Two-tailed unpaired *t*-test, Wilcoxon Matched-Pairs Signed-Ranks Test were applied to compare other mean values. Bivariate correlation analysis was performed to understand the relations between parameters from swimming exercise and the biological indices of the swimming training. The correlation coefficient was expressed as Pearson’s r. The number of independent experiments and the statistical tests employed are indicated in the respective figure legends. *p* < 0.05 was considered statistically significant.

## 3 Results

### 3.1 Multi-target trajectory tracking revealed considerable individual differences in swimming behavior

Real-time video analytics was used to monitor the locomotor activity of swimming mice using a custom-established rodent swimming tank (Figure 1A). The tank was able to simultaneously monitor up to 20 rodents during one session of swimming training. Here, 45 mice were subjected to swimming exercise, with 8–10 mice in each session. As shown in Figure 1B, the trajectory of each mouse during swimming (60 min) was recorded, and the distance and velocity were calculated (Supplementary Video S1). The total distance swam by each mouse in one session of exercise varied considerably, with a maximal distance of 525 m and a minimum distance of 72 m (Figures 1C, D). Gaussian fitting to total distance histogram revealed that the total distance was centered at 296 m with a full width at half maximum (FWHM) of 174 m (Figure 1E). The individual difference was also observed in the average velocity, with a maximal velocity of 8.8 m/min and a minimum velocity of 1.2 m/min (Figures 1F–H). Gaussian fitting to average velocity histogram revealed that average velocity was centered at 5 m/min with a FWHM of 2.68 m/min (Figure 1E). For the temporal changes of velocity during swimming, the maximum velocity was observed at the beginning of swimming, and then the velocity maintained relatively constant after about 10 min, indicating an acute adaptation (Figures 1I, J). These results suggested that trajectory tracking revealed considerable individual differences in behavior during swimming.

### 3.2 Different trajectory patterns during swimming exercise

**FIGURE 2:**
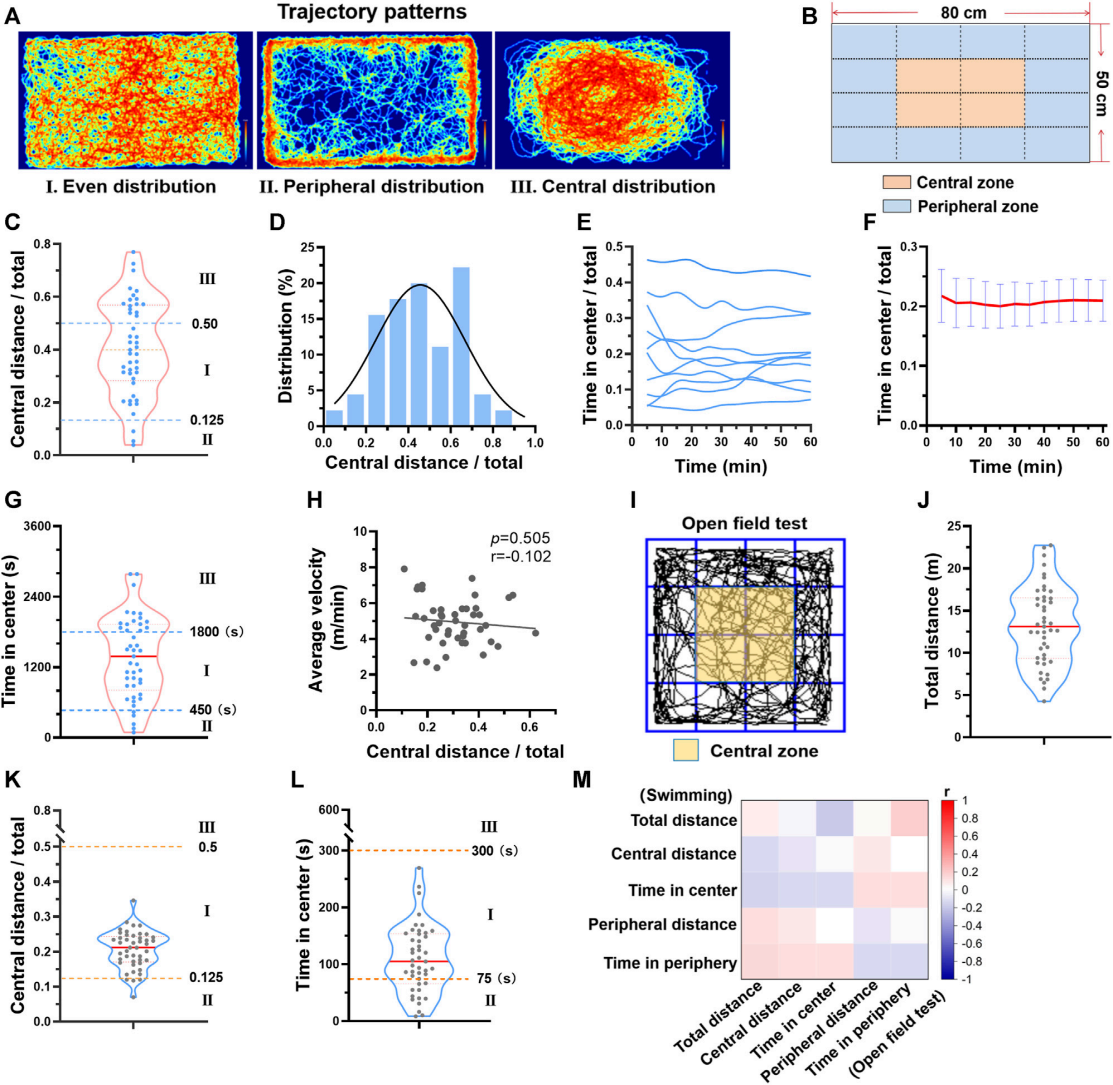
Different trajectory patterns during swimming exercise. **(A)** There are 3 typical trajectory patterns during swimming. Typical trajectory plots of 3 patterns are shown. **(B)** The swimming zone was divided into central zone and peripheral zone. **(C)** The distribution of central distance/total distance of 45 mice. **(D)** Central distance/total distance histogram of 45 mice. **(E)** Typical temporal changes of time in center of 10 mice during one session of swimming training. **(F)** Average time in center along with time during one session of swimming training (*n* = 10). **(G)** The distribution of time in center of 45 mice. **(H)** The relation between central distance/total distance and average velocity (*n* = 45). **(I)** The open field test zone was divided into central zone and peripheral zone. **(J–L)** The distributions of total distance, central distance/total distance, and time in center from open field test (*n* = 45). **(M)** Correlation analysis suggested that parameters from open field test were not correlated with parameters from swimming exercise (all *p* values < 0.05). n is indicated in each panel.

Besides the individual differences in distance and velocity, we also observed a difference in the swimming zone each mouse favored. There are 3 typical patterns, even distribution, peripheral distribution, and central distribution (Figure 2A). Next, the swimming zone was divided into central zone and peripheral zone (Figure 2B), and the time spent and the distance swam at different zones were calculated. Regarding to central distance/total distance during one session of swimming, 57.7% of mice exhibited even distribution and 35.6% of mice exhibited central distribution, while only 6.7% of mice exhibited peripheral distribution (Figure 2C). The central distance/total distance histogram was centered at 0.41 (0.25 represents an even distribution), suggesting that mice preferred swimming at the center zone (Figure 2D). Although the trajectory pattern varied among mice, it maintained temporal stability for each mouse during one session of swimming training (Figures 2E, F). Regarding to time in center, a similar distribution was observed (Figure 2G). In addition, there was no linear relation between central distance/total distance and average velocity (Figure 2H), suggesting that the trajectory pattern was not likely to affect velocity during swimming.

Given that the trajectory pattern analysis is usually used in behavior experiments, we next compared the trajectory pattern during swimming with that of the open field test. The same parameters were calculated (Figure 2I). Mice exhibited different behavior in two tests (Figure 2J-L). For example, mice favored peripheral zone in the open field test, but central zone in swimming (Figure 2K). Correlation analysis also suggested that parameters from open field test were not correlated with parameters from swimming exercise (Figure 2M). Thus, the trajectory pattern in swimming is not likely to indicate exploratory activity and anxiety which are reflected by open field test.

### 3.3 Swimming behavior adaptations to long-term exercise varied among mice

**FIGURE 3:**
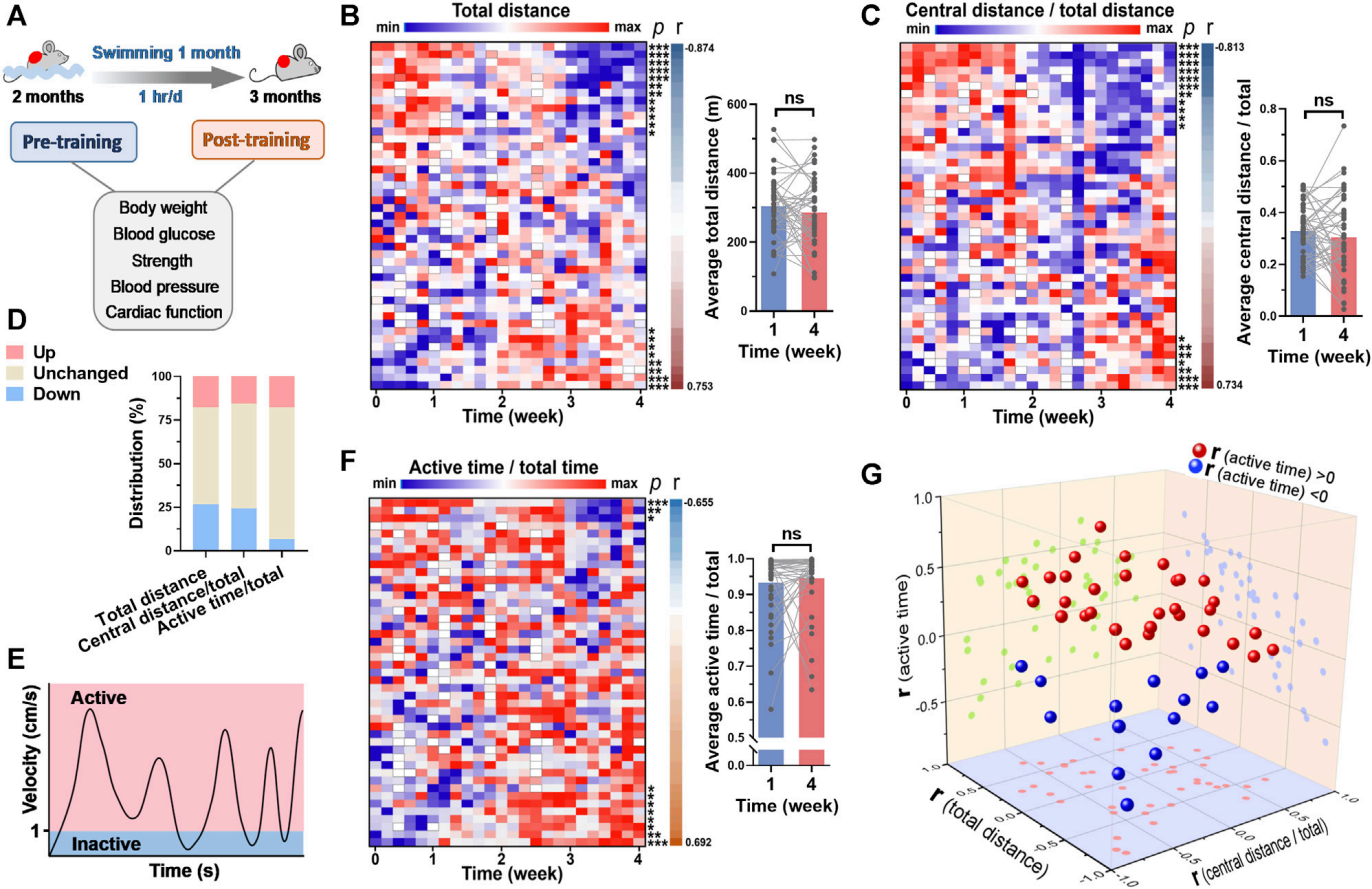
Swimming behavior was hardly changed by long-term exercise in general. **(A)** Mice were subjected to swimming training for 1 month, and the basicphysiological indices were detected before and after training. **(B)** A heatmap showing the changes of total distance in each swimming session along with time, with each line representing a mouse. Quantified total distance (first week vs. last week) was shown in right. r and *p* values are from the correlation analyses between total distance and time for each mouse. **(C)** The changes of central distance/total distance in each swimming session along with time. **(D)** The distribution of 3 parameters for their changes along with time. **(E)** Definition of inactive and active swimming statuses. **(F)** The changes of active time/total time in each swimming session along with time. **(G)** 3D plot illustrating the r values of 3 parameters for each mouse. r values are from **(B,C)** and **(F)**. *n* = 45. *, *p* < 0.05; **, *p* < 0.01; ***, *p* < 0.001.

The mice were subjected to swimming training for 1 month, and the cardiac function and blood pressure were detected before and after training (Figure 1A). The swimming behavior adaptations to long-term training were analyzed. For total distance swam by mouse in each session of training, it was decreased along with time in 12 mice (26.7%) and increased along with time in 7 mice (15.6%) (Figure 3B). For central distance/total distance, it was decreased along with time in 11 mice (24.4%) and increased along with time in 7 mice (15.6%) (Figures 3C, D). In addition, we introduced a new parameter to assess whether the mouse is active during swimming. The status when the velocity was <1 cm/s was defined as inactive, otherwise active (Figure 3E). For active time/total time, 3 mice (6.7%) exhibited a decrease along with time, and 7 mice (15.6%) showed an increase along with time (Figures 3F, G). In general, the swimming behavior, including total distance, central distance/total, time in center/total, and active time/total showed no changes in the last week of training compared with that in the first week of training (Figures 3B, C, F, Supplementary Figure S1). These results suggested that swimming behavior is hardly changed in general.

### 3.4 Long-term swimming training induced improvements in cardiac function and blood pressure

**FIGURE 4:**
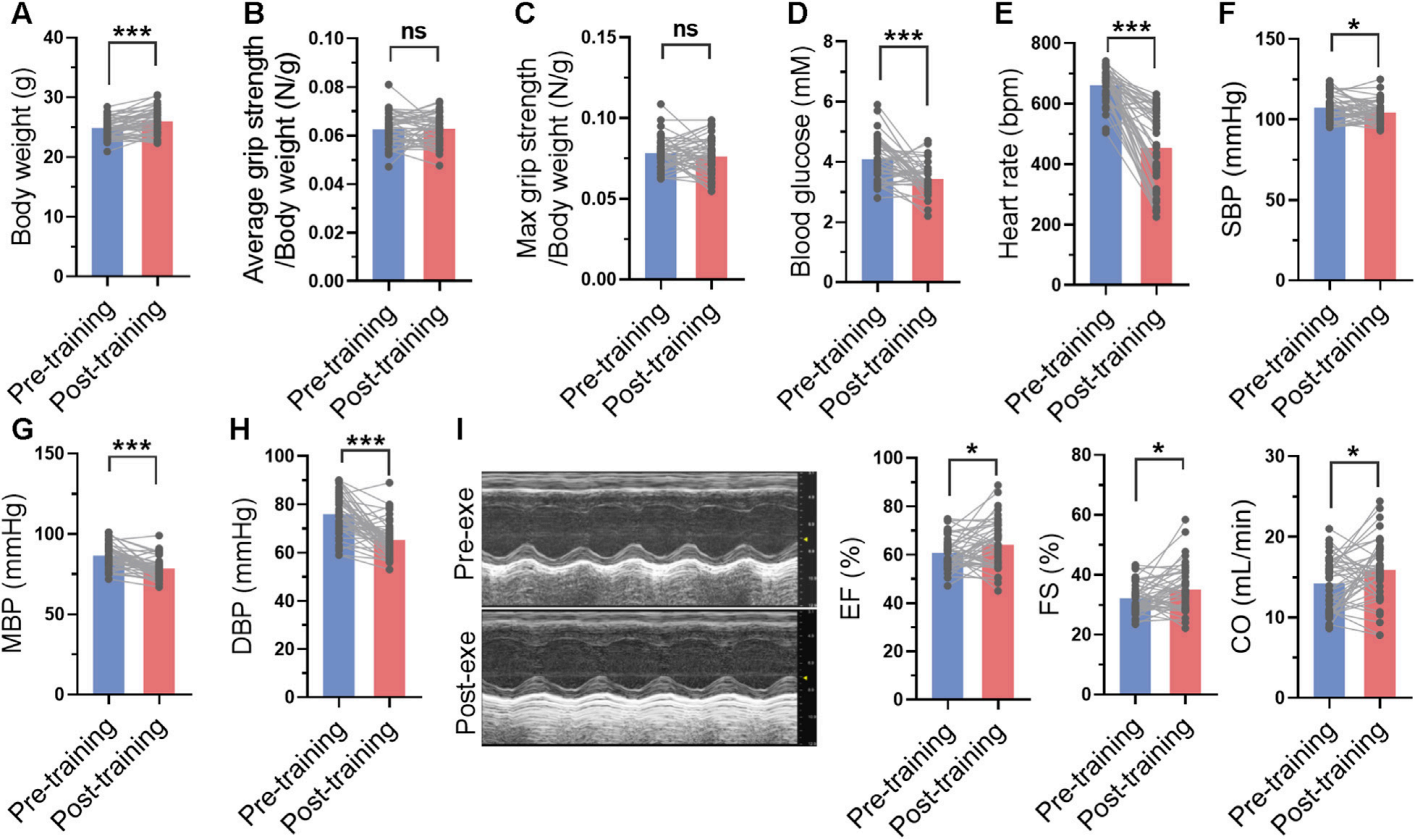
Long-term swimming training induced improvements in cardiac function and blood pressure. **(A)** Body weight before and after swimming training. **(B–D)** Average **(B)** and maximal **(C)** grip strength before and after swimming training. **(E–H)** Heart rate **(E)**, systolic blood pressure (SBP) **(F)**, mean blood pressure (MBP) **(H)**, and diastolic blood pressure (DBP) **(H)** before and after swimming training. **(I)** Cardiac function before and after swimming training. Typical M-mode images were shown in left, and quantified ejection fraction (EF), fractional shortening (FS) and cardiac output (CO) were shown in right. *n* = 45. *, *p* < 0.05; ***, *p* < 0.001.

The blood glucose, blood pressure and cardiac function were measured before and after 1-month swimming training. After swimming training, the body weight of mice was increased (Figure 4A). Although swimming training did not affect average grip strength and maximal grip strength of mice, it decreased blood glucose by 16% (Figures 4B–D). In addition, swimming training decreased heart rate, and systolic and diastolic blood pressure (Figures 4E–H). It also improved cardiac function, as evidenced by increased ejection fraction, fractional shortening, and cardiac output (Figure 4I). These results suggested that long-term swimming training induced improvements in cardiac function and blood pressure.

### 3.5 Swimming behavior was associated with cardiac function post swimming training

**FIGURE 5:**
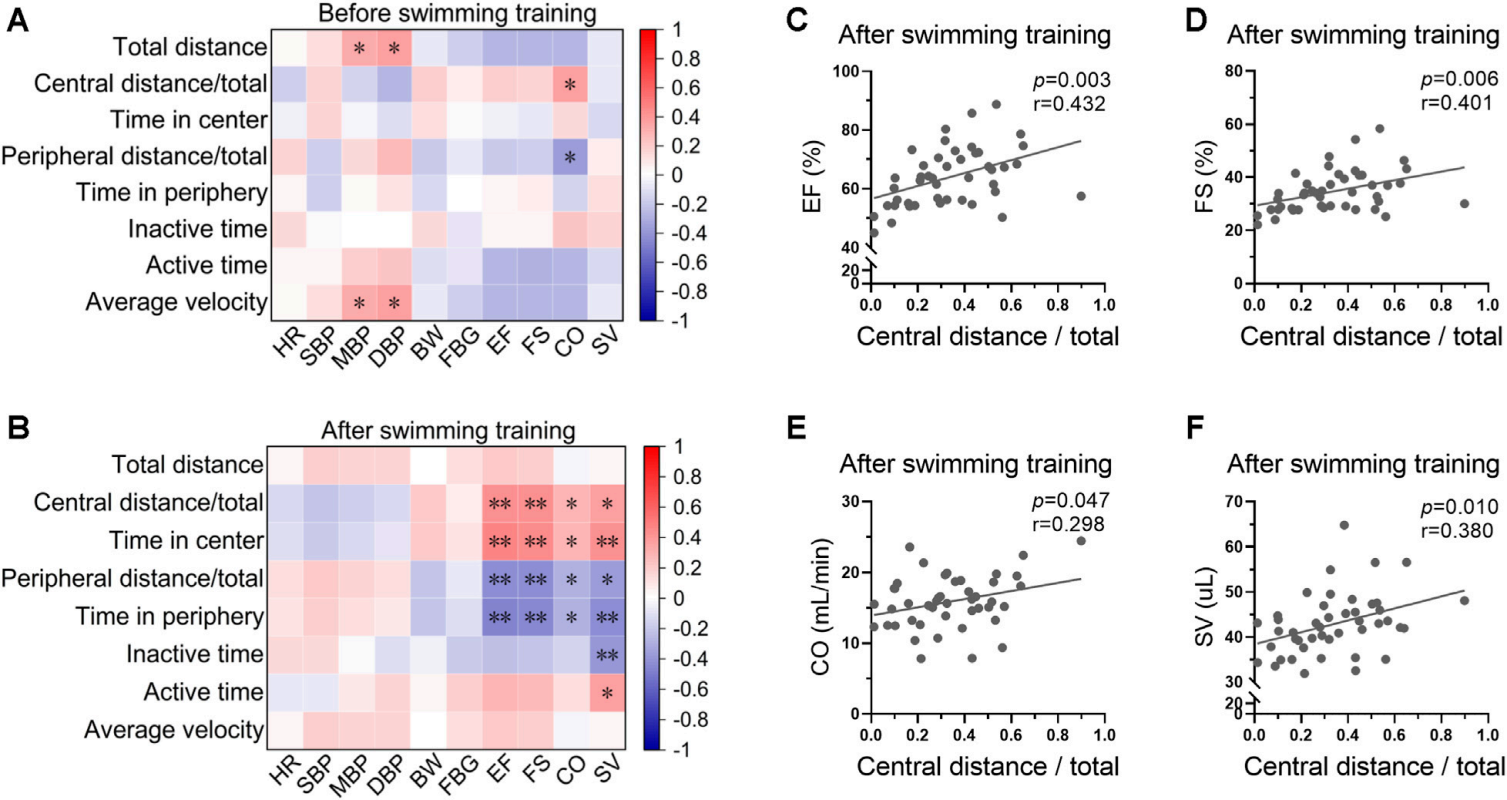
Swimming behavior was associated with cardiac function post swimming training. **(A)** Heatmap showing the correlations between swimming behavior and biological indices at the beginning of swimming training. **(B)** Heatmap showing the correlation between swimming behavior and biological indices post swimming training. **(C–F)** The correlations between central distance/total and EF **(C)**, FS **(D)**, CO **(E)**, and SV **(F)** post swimming training. *n* = 45. *, *p* < 0.05; **, *p* < 0.01.

The correlations between swimming behavior and biological indices before and after swimming training were analyzed. There was a weak correlation between swimming behavior and biological indices at the beginning of swimming training (Figure 5A, Supplementary Figure S2). For instance, blood pressure was positively correlated with total distance and average velocity before exercise training, indicating a physiological significance of blood pressure in regulation of swimming behavior. However, the correlation between swimming behavior and biological indices was stronger post swimming training (Figure 5B, Supplementary Figure S2). Specifically, cardiac function showed positive correlations with central distance/total and time in center, and negative correlations with peripheral distance/total and time in periphery post swimming training (Figures 5C–F), indicating a potential role of swimming behavior in indicating the biological effects of exercise on cardiac function.

### 3.6 Mice preferring swimming at the central zone showed more obvious exercise-induced benefits in blood pressure and cardiac function

**FIGURE 6:**
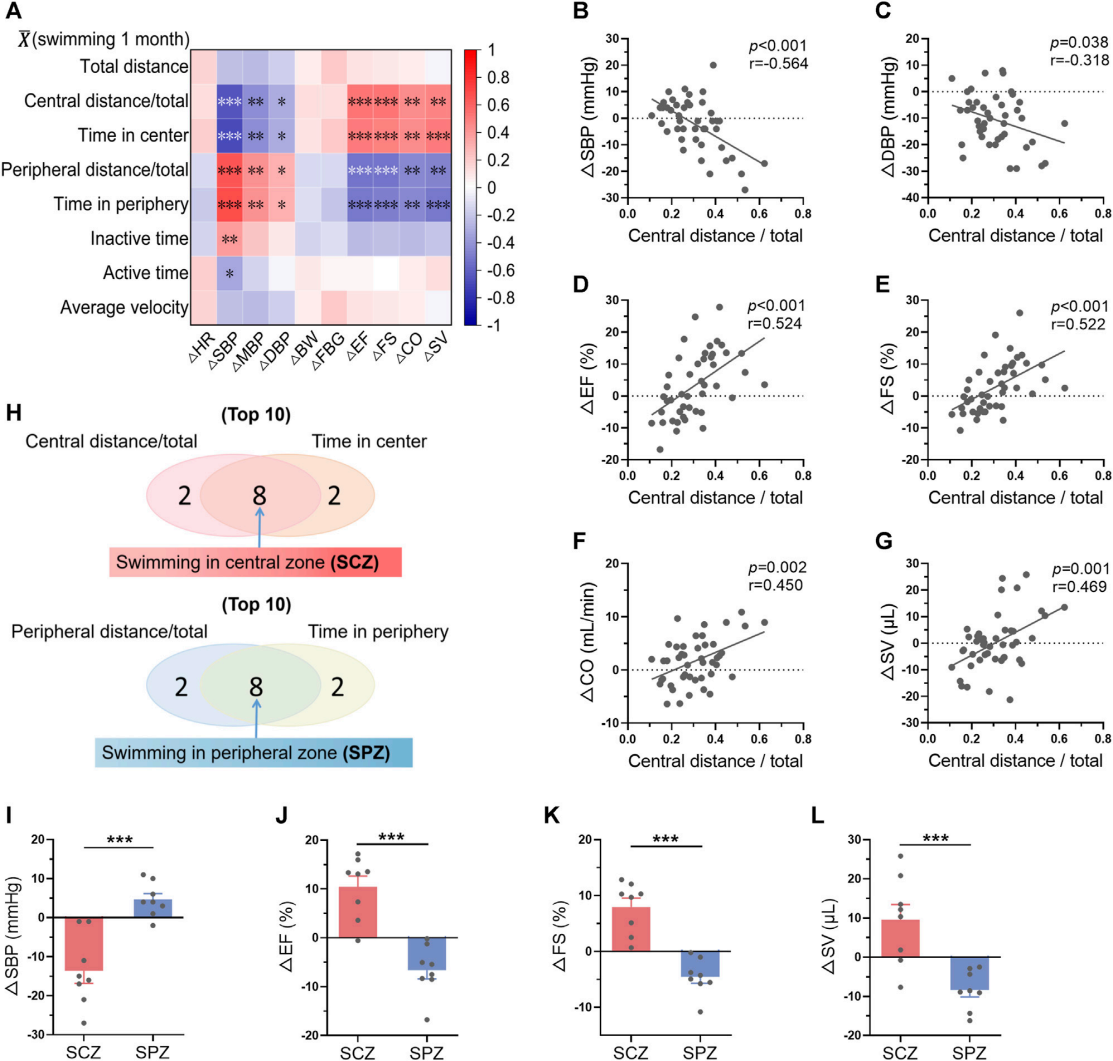
Mice preferring swimming at the central zone showed more obvious exercise-induced benefits in blood pressure and cardiac function. **(A)** Correlation between average swimming behavior indices within exercise training and the changes of biological indices-induced by exercise training (ΔR = Rpost-training-Rpre-training). **(B–G)** Correlations between average central distance/total within 1-month training and ΔSBP **(B)**, ΔDBP **(C)**, ΔEF **(D)**, ΔFS **(E)**, ΔCO **(F)**, and ΔSV **(G)**. **(H)** The top 8 mice which preferred swimming at the central zone (SCZ) and the top 8 mice which preferred swimming at the peripheral zone (SPZ) were identified according to the average swimming behaviors within 1-month training. **(I–L)** ΔSBP **(I)**, ΔEF **(J)**, ΔFS **(K)**, and ΔSV **(L)** in SCZ and SPZ mice. *n* = 45 in **(A–G)**, and 8 in **(I–L)**. *, *p* < 0.05; **, *p* < 0.01; ***, *p* < 0.001.

The changes of biological parameters (ΔR = Rpost-training-Rpre-training) were defined as the effects of long-term exercise. The correlations between average swimming behavior indices within 1-month training and ΔRs were then analyzed. The results displayed a strong correlation between swimming area preference and exercise-induced biological effects, including blood pressure and cardiac function (Figure 6A). Specifically, average central distance/total and time in center within 1-month swimming training are negatively correlated with the changes of blood pressure, and positively correlated with the changes of cardiac function, indicating central zone swimming preference is associated with more obvious exercise-induced benefits on blood pressure and cardiac function (Figures 6A–G). In contrast, peripheral distance/total and time in peripheral within 1-month swimming training are positively correlated with the changes of blood pressure, and negatively correlated with the changes of cardiac function (Figure 6A, Supplementary Figure S3).

Among the 45 mice, the top 8 mice which preferred swimming at the central zone and the top 8 mice which preferred swimming at the peripheral zone were identified according to the average swimming behaviors within 1-month training (Figure 6H). Interestingly, these 2 groups of mice exhibited distinct biological outcomes induced by swimming training (Figures 6I–L). For example, swimming training decreased blood pressure and increased cardiac function in mice preferring swimming at the central zone, while blood pressure was increased and cardiac function was decreased in mice preferring swimming at the peripheral zone (Figures 6I–L). These results suggested that mice preferring swimming at the central zone show more obvious exercise-induced benefits in blood pressure and cardiac function.

### 3.7 Mice preferring swimming at the central zone showed milder stress during exercise

**FIGURE 7:**
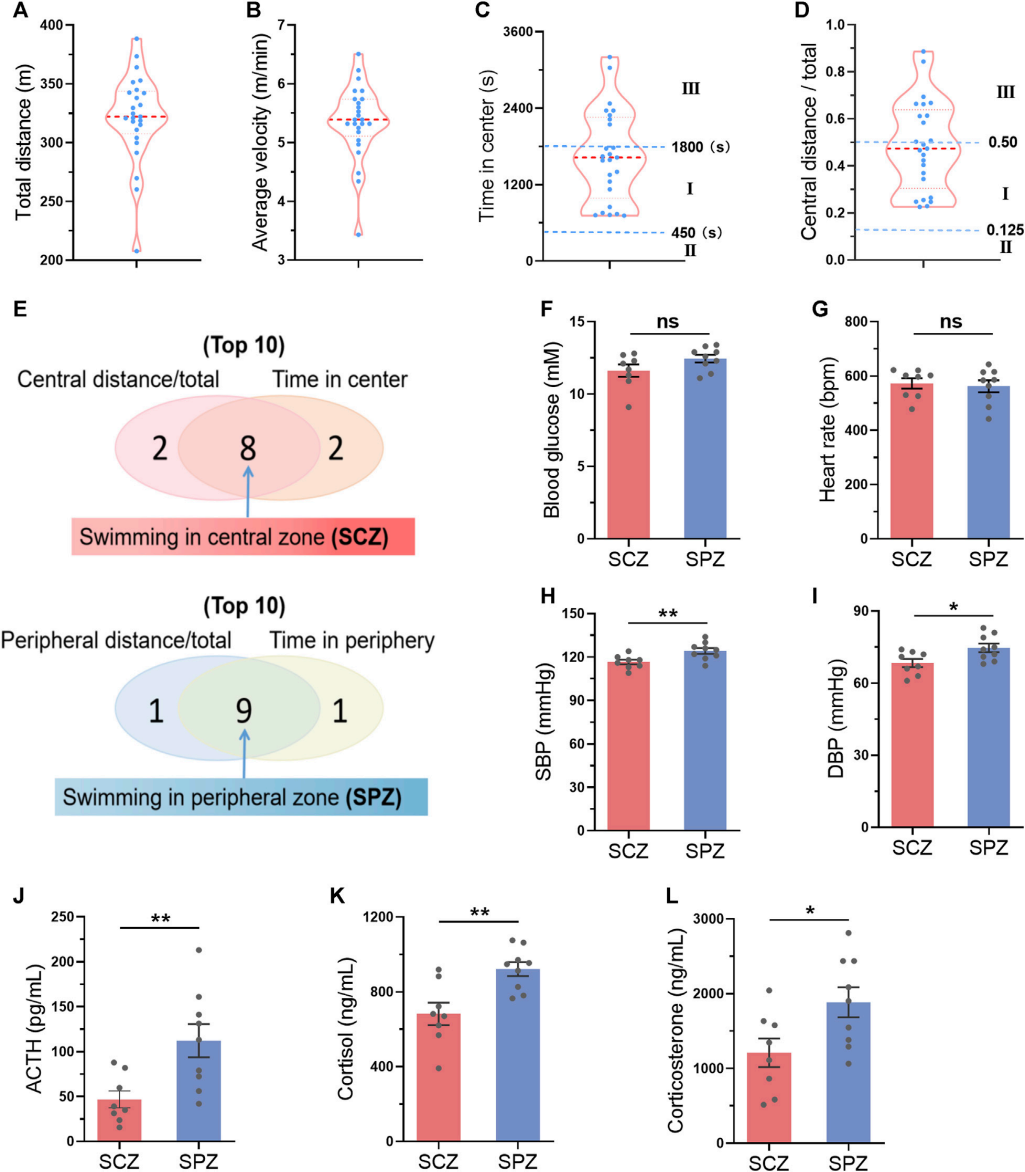
Mice preferring swimming at the central zone showed milder stress during exercise. **(A–D)** The distribution of total distance **(A)**, average velocity **(B)**, time in center **(C)**, and central distance/total **(D)** in 25 mice after a single bout of swimming exercise. **(E)** The top 8 mice which preferred swimming at the central zone (SCZ) and the top 9 mice which preferred swimming at the peripheral zone (SPZ) were identified during one bout of swimming exercise. (FL) Blood glucose **(F)**, heart rate **(G)**, SBP **(H)**, DBP **(I)**, and circulating ACTH **(J)**, cortisol **(K)**, and corticosterone **(L)** in SCZ and SPZ mice immediately after one bout of exercise. *n* = 25 in **(A–D)**, and eight to nine in **(F–L)**. *, *p* < 0.05; **, *p* < 0.01.

We hypothesized that mice preferring swimming at the central zone may indicate a smaller stress during exercise. We next tested the activation of hypothalamic–pituitary–adrenal axis, the major component of the neuroendocrine network responds to stress (Leistner and Menke, 2020), immediately after one bout of exercise in parallel experiments. A total of 25 mice were subjected to swimming exercise, and the behavior of the mice were recorded. As shown in Figures 7A–D, the behaviors of the mice varied. Then, the top 8 mice which preferred swimming at the central zone and the top 9 mice which preferred swimming at the peripheral zone were identified during one bout of swimming exercise (Figure 7J). Mice preferring swimming at the peripheral zone showed higher activation of hypothalamic–pituitary–adrenal axis, as evidenced by increased blood pressure, and elevated circulating concentrations of ACTH, cortisol, and corticosterone, although differences in blood glucose and heart rate were not detected (Figures 7F–L). These results suggested that mice preferring swimming at the central zone face milder stress.

### 3.8 Swimming behavior adaptations indicated long-term exercise-induced cardiac benefits

**FIGURE 8:**
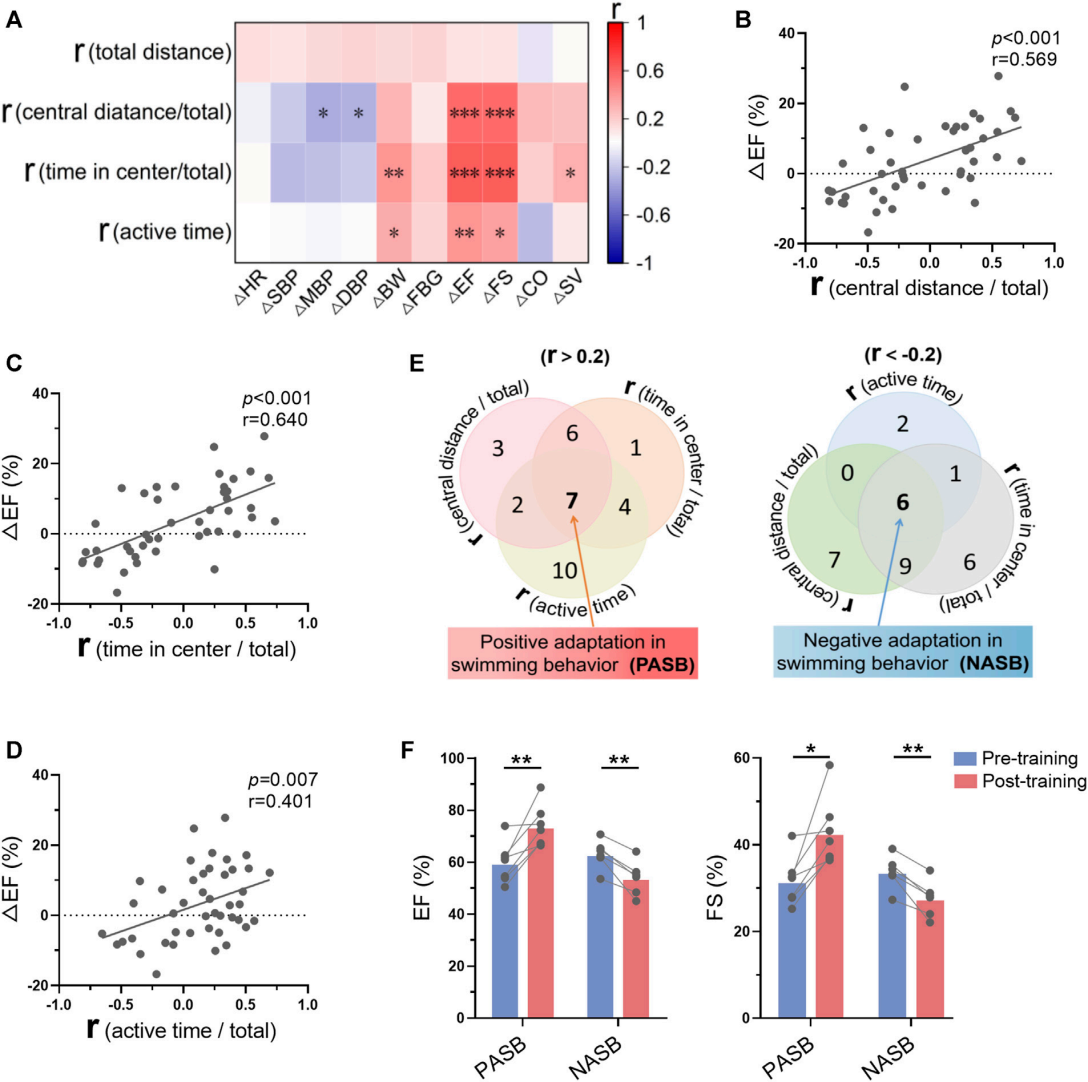
Swimming behavior adaptations indicated long-term exercise-induced cardiac benefits. **(A)** Correlation between swimming behavior adaptations (r values were calculated in Figure 3) and the changes of biological indices-induced by exercise training. **(B–D)** Correlations between ΔEF and r (central distance/total) **(B)**, r (time in center/total) **(C)**, r (active time/total) **(D)**. **(E)** Seven mice were identified as trainable mice (positive swimming behavior adaptations) and 6 mice were identified as untrainable mice (negative swimming behavior adaptations) according to 3 r values. **(F)** Exercise training increased cardiac function in trainable mice, while decreasing cardiac function in untrainable mice. *n* = 45 in **(A–D)**, and 6-7 in **(F)**. *, *p* < 0.05; **, *p* < 0.01; ***, *p* < 0.001.

Mice were trained for 1 month, and we observed that only a part of mice were trainable (positive swimming behavior adaptations), regarding to swimming behavior adaptations to exercise training analyzed in Figure 3. To understand the association between the changes of swimming behavior along with training and exercise-induced biological changes, we analyzed the correlation between these two groups of parameters. As shown in Figure 8A, there are strong correlations between the r values of swimming behavior and the changes of cardiac function (Supplementary Figure S4). Specifically, r values in central distance/total, time in center/total, and active time were positively correlated with Δ ejection fraction (Figures 8B–D), indicating that trainable mice in swimming behavior are associated with better exercise-induced benefits in cardiac function. Then, 7 mice were identified as trainable mice and 6 mice were identified as untrainable mice (negative swimming behavior adaptations) according to 3 r values (Figure 8E). Interestingly, exercise training increased cardiac function in trainable mice, while decreased cardiac function in untrainable mice (Figure 8F). These results suggested that mice with positive swimming behavior adaptations during training show more obvious exercise-induced benefits in cardiac function.

## 4 Discussion

Although swimming is one of the mostly used models of rodent exercise, concerns have been raised regarding to its limitations. Here, we established a multi-target system to monitor the individualized rodent behavior during swimming. Based on the analyses of swimming behavior in 45 mice during long-term exercise training, we found that swimming behavior varies among mice, which characteristics and adaptations indicate long-term exercise-induced cardiovascular adaptations. Specifically, mice which preferred swimming in central zone or were trainable in behavior during 1-month training showed more obvious exercise-induced benefits in blood pressure and cardiac function. Mechanistically, a centralized swimming behavior indicated a smaller stress during exercise. These findings highlight the significance of swimming behavior monitoring in exploring the underlying mechanisms of exercise-induced benefits when rodent swimming is used as a model of exercise.

Swimming behavior has long been used in behavior studies, including the forced swim test and Morris water maze, although these tests are different from swimming exercise (Dawson and Horvath, 1970; Yan et al., 2010). Forced swim test is used to assess antidepressant-like activity in rodents (Porsolt et al., 1977; Slattery and Cryan, 2012). Morris Water Maze is used to assess hippocampus-dependent spatial navigation and reference memory (Vorhees and Williams, 2006; Othman et al., 2022). These well-established methods and their applications suggest that the rodent behavior in water reflects rodent status. Here, we established a new method to monitor individualized swimming behavior using a custom-developed multi-target system during swimming exercise. Swimming behavior of each rodent during exercise, including trajectory, distance, velocity, status, and area preference, is recorded and analyzed. It is obvious that the swimming behavior in swimming exercise is different from that in forced swim test and Morris water maze which induce higher stress in rodents. Swimming exercise is more enjoyable due to the water temperature, space, and group activity (Hastings et al., 2022). We compared the behavior of mice in swimming and open field test, and found that that behaviors in open field test and swimming exercise were not correlated, reinforcing the notion that rodent behavior in swimming exercise is different from that in other behavior tests.

Based on the data from 45 mice during one session of swimming exercise, it is surprising that the behaviors of mice vary largely. For example, the total distance swam by each mouse in one session of exercise varied from a maximal distance of 525 m to a minimum distance of 72 m, indicating the workloads of mice were very different. Mounting evidence has shown that the effects of exercise are largely dependent on the intensity of exercise (Goto et al., 2003; Kemi et al., 2005; Sattelmair et al., 2011). It is generally believed that a higher intensity of exercise is accompanied with a better outcome (Arem et al., 2015; Paluch et al., 2023). Thus, the individual differences in swimming behavior may underly the variations in exercise-induced effects in animal studies. Surprisingly, we only observed a weak association between total distance and blood pressure before swimming training, without any association between total distance and long-term exercise-induced biological changes, indicating that other factors in swimming behavior may play a more important role. In fact, we observed a strong correlation between area preference in swimming behavior and long-term exercise-induced biological effects. Specifically, mice preferring swimming at the central zone showed more obvious exercise-induced benefits in blood pressure and cardiac function, and long-term exercise even induced adverse effects on blood pressure and cardiac function in mice preferring swimming at the peripheral zone. These results suggested that swimming area preference rather than workload is more important in determining the biological effects of long-term exercise. Importantly, we found that a centralized swimming behavior indicates a smaller stress during exercise, as evidenced by a milder activation of hypothalamic–pituitary–adrenal axis, while mice preferring swimming at the peripheral zone show a higher stress. These stress responses in mice preferring swimming at the peripheral zone may counteract the positive effects of exercise and potentially lead to long-term adverse effects. These results suggested that the stress during swimming underlies the correlation between swimming behavior and cardiovascular outcomes induced by long-term exercise.

It is well established that long-term exercise induces profound adaptations in cardiovascular system (Trivedi and Epstein, 2011; Wu et al., 2022; Perry et al., 2023). Consistently, long-term exercise decreased blood pressure and increased cardiac function as observed in our experiments. However, whether swimming behavior can be trainable is unknown. Here, we showed that swimming behavior remained relatively constant during one session of swimming. In addition, 1-month swimming training showed no significant improvements in active time, time in center/total, central distance/total, and total distance in general. These results suggested that the swimming behavior in mice is hardly trainable. Similarly, the swimming skill is not likely to be improved if there is no intended training in humans (Smith et al., 2002; Amara et al., 2023). However, some mice exhibited positive swimming behavior adaptations to long-term training. For example, 13% of mice showed improvements along with time in total distance and active time. Conversely, long-term training induced negative swimming behavior adaptations in some mice, indicating a large variation among mice. More importantly, correlation analyses revealed that behavior adaptations indicate the biological outcomes of exercise. Specifically, trainable mice whose behavior became more active during swimming exhibited cardiac benefits induced by exercise, while untrainable mice showed decreases in cardiac function after swimming training. It should be noted that the swimming behavior rather than the changes of swimming behavior determines the effects of swimming training. We analyzed the correlation between swimming behavior and cardiac function before swimming training, and there was a weak correlation between swimming behavior and biological indices at the beginning of swimming training, indicating a weak association between swimming behavior with cardiovascular function in mice without swimming training. Swimming behavior is more likely to be a determinant of swimming training-induced cardiovascular adaptations.

Taken together, we established a new method for monitoring individualized swimming behavior during swimming exercise, and found that swimming behavior varies largely among mice and swimming behavior indicates the biological outcomes of long-term exercise training. Specifically, mice which prefer swimming in central zone exhibit better outcomes in cardiac function and blood pressure post long-term exercise due to a milder stress during exercise. These results highlight the biological significance of swimming behavior in animal studies, which should be monitored to illustrate individual differences.

## Data Availability

The original contributions presented in the study are included in the article/Supplementary Material, further inquiries can be directed to the corresponding authors.
