# Supplementary material for: Swimming behavior indicates stress and adaptations to exercise
Source: Front Physiol. 2024 Feb 26;15:1357120. doi: 10.3389/fphys.2024.1357120 (PMC10925659; doi:10.3389/fphys.2024.1357120)
Supplement: Supplementary file 1 [file DataSheet1.PDF]

Supplementary Material

Swimming behavior indicates stress and adaptations to exercise

Sen Yu[1]†, Lantao Liu[2]†, Yong Liu[1,*], Xing Zhang[1,4,*]

*** Correspondence:** Xing Zhang, Ph.D., 169 Changlexi Road, Xi'an 710032, China. Tel/Fax: 86-29-84711275. E-mail: zhangxing@fmmu.edu.cn or Yong Liu, Ph.D., 169 Changlexi Road, Xi'an 710032, China. Tel/Fax: 86-29-84711211. E-mail: liuyong@fmmu.edu.cn

1 Supplementary Figures

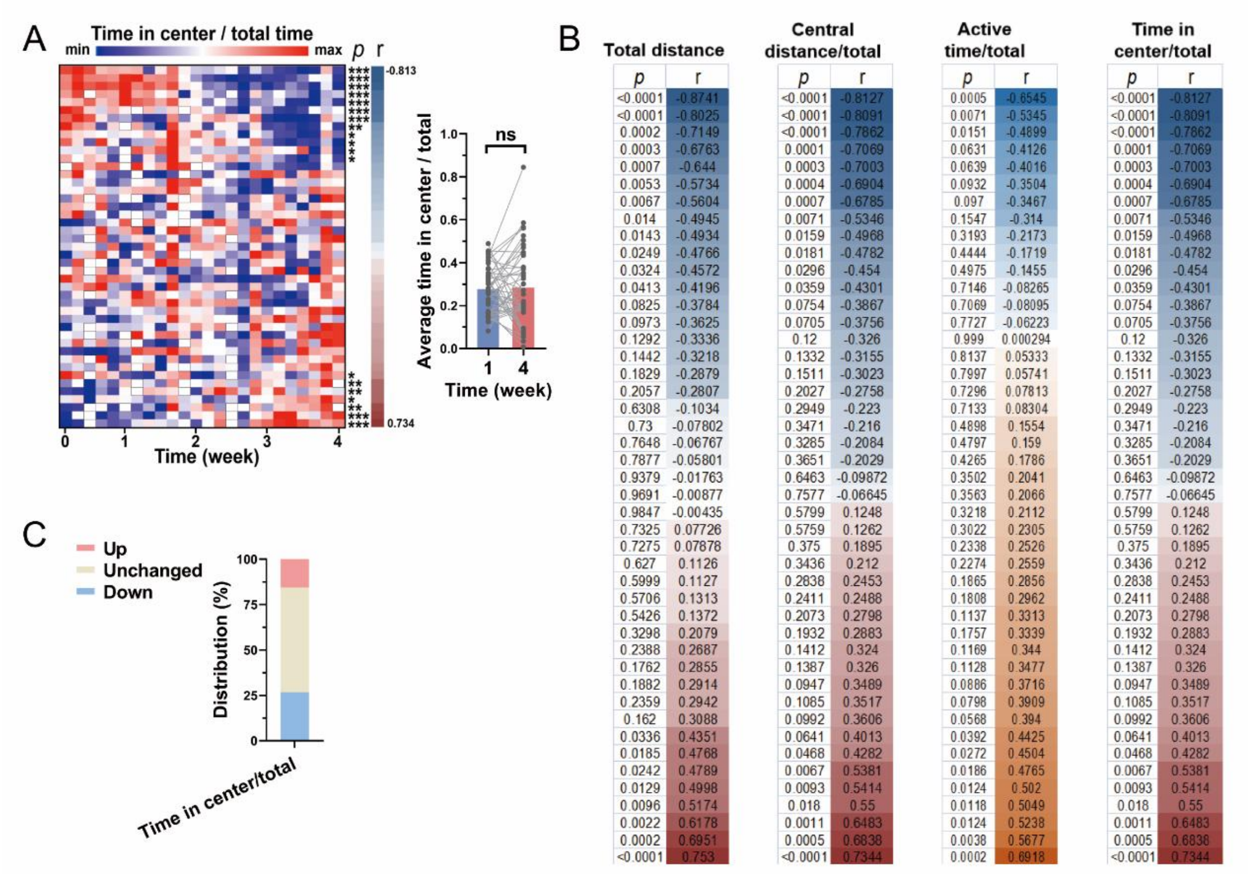

Figure S1. Swimming behavior adaptations to long-term exercise

A. The changes of time in center along with time. A heatmap of time in center/total time with each line representing a mouse was shown in left, and quantified result of time in center/total time (first week vs. last week) was shown in right. r and p values are from the correlation analyses between time in center and time for each mouse. B. r and p values for total distance, central distance/total, active time, and time in center. C. The distribution of time in center for its changes along with time. n=45

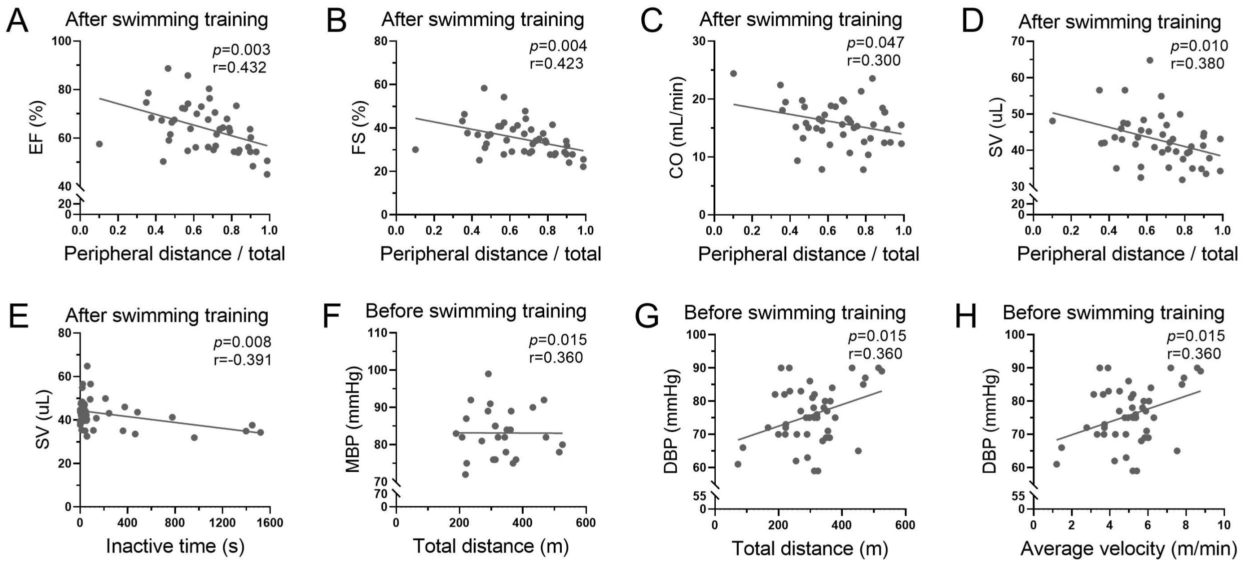

Figure S2. Swimming behavior was associated with cardiac function post swimming training

A-D. The correlations between peripheral distance/total with EF (A), FS (B), CO (C), and SV (D) post swimming training. E. The correlations between inactive time with SV post swimming training. F-G. The correlations between total distance with MBP (F) and DBP (G) before swimming training. H. The correlations between average velocity with DBP before swimming training. n=45.

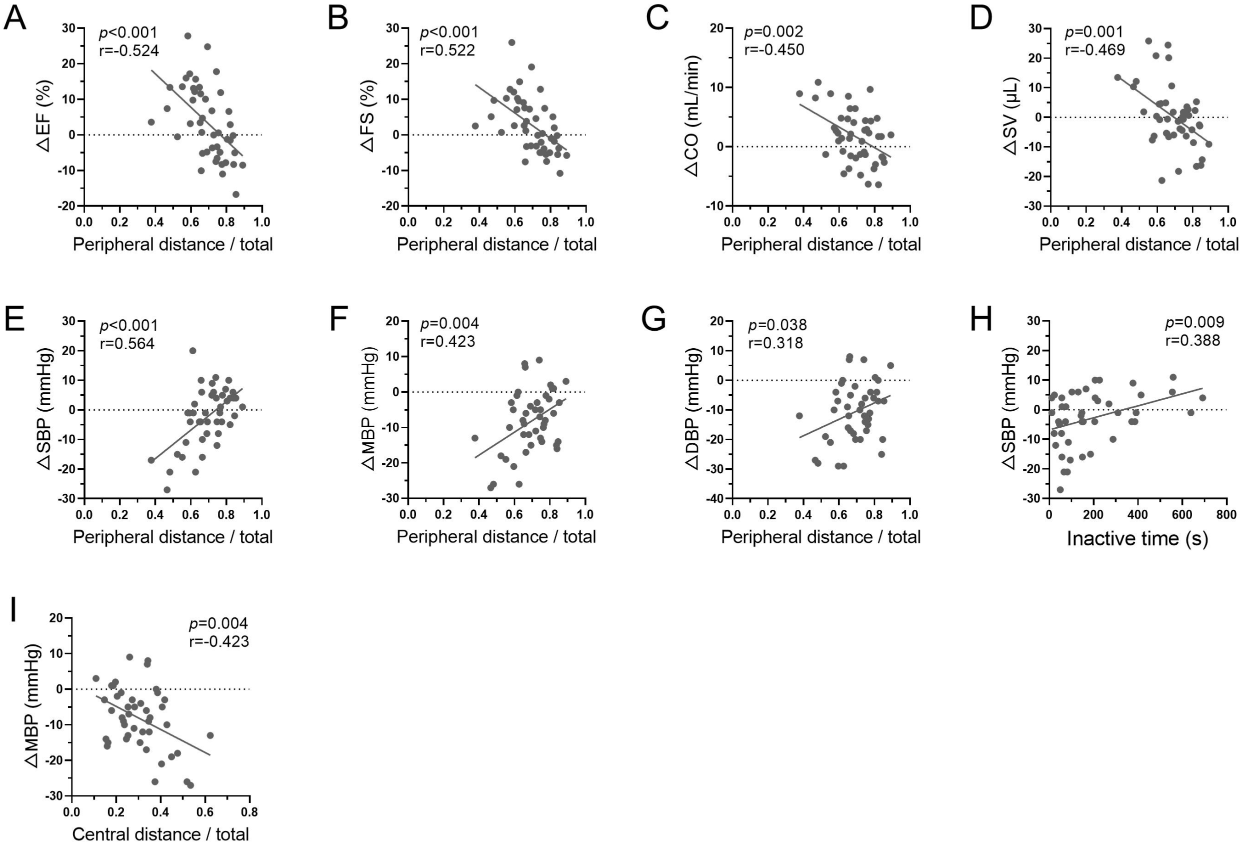

Figure S3. Mice preferring swimming in the central zone showed more obvious exercise-induced benefits in blood pressure and cardiac function

A-G. Correlations between average peripheral distance/total within 1-month training and ΔEF (A), ΔFS (B), ΔCO (C), ΔSV (D), ΔSBP (E), ΔMBP (F), and ΔDBP (G). H. Correlations between average inactive time within 1-month training and ΔSBP. I. Correlations between average central distance/total within 1-month training and ΔMBP. n=45.

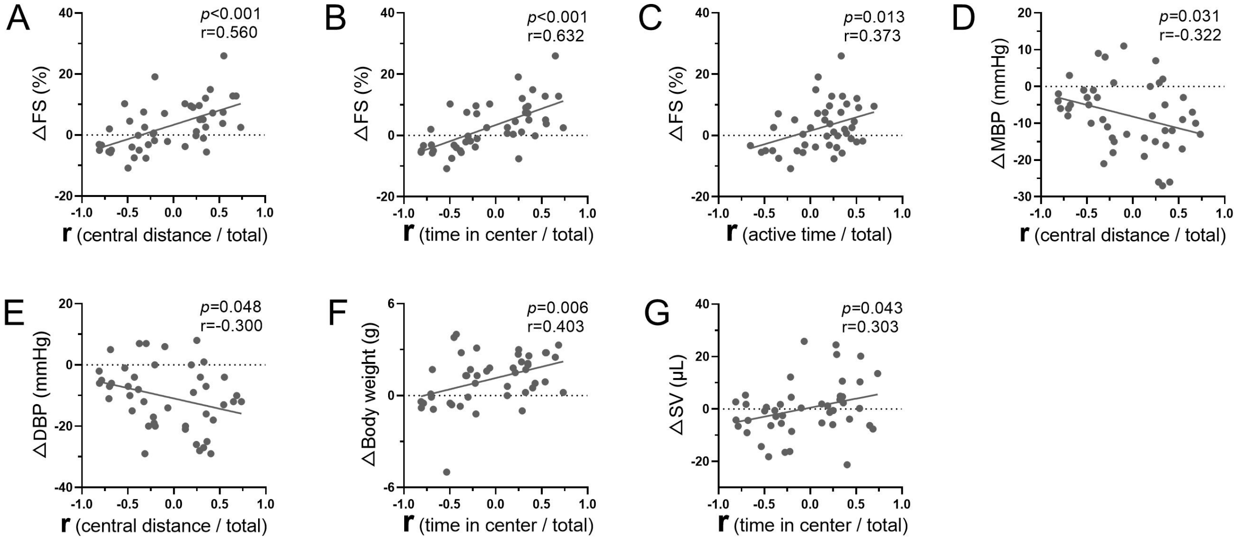

Figure S4. Swimming behavior adaptations indicated long-term exercise-induced cardiac benefits

A-C. Correlations between ΔFS and r (central distance/total) (A), r (time in center/total) (B), and r (active time/total) (C). D-E. Correlations between ΔMBP (D), ΔDBP (E) and r (central distance/total). F-G. Correlations between Δbody weight (F), ΔSV (G) and r (time in center/total). n=45.
